# Delving into Acromegaly

**DOI:** 10.3390/jcm12041654

**Published:** 2023-02-19

**Authors:** Sylvère Störmann, Katharina Schilbach

**Affiliations:** Medizinische Klinik und Poliklinik IV, Klinikum der Universität München, Ludwig-Maximilians-Universität München, Ziemssenstr. 5, 80336 München, Germany

Acromegaly is a rare and disabling disease with some distinct and striking clinical features that have fascinated (and frightened) laypeople and medical experts alike throughout history [1]. Its prevalence is around 50 to 80 patients per million people worldwide [2], with a regional variability e [3]. Since, in many cases, the disease is detected late or not at all, these figures may be an underestimate of the actual cases. Acromegaly is registered in the Orphanet database under code 963. Despite (or maybe because of) its rarity, there is a very active international research community devoted to unravelling its secrets. While web searches for the word “acromegaly” (as a surrogate parameter for public interest) remain quite stable over the years, the number of PubMed-listed publications containing the word stem “acromegal*” in their title has doubled over the past 20 years. Notably, a few studies with these titles were recently published in the *Journal of Clinical Medicine* and its sibling journals, covering all aspects of the disease, from basic science and diagnostics to current and possible future management.

Acromegaly is caused by an excess of growth hormone (GH), in most cases due to a GH-secreting pituitary adenoma with an abundance of somatotroph cells [4]. Astonishingly, the pathogenesis leading to these tumors is still not fully understood. However, several germline and somatic mutations have been discovered that lead to acromegaly or gigantism [5]. In a recent review, Yamamoto and Takahashi comprehensively elaborate on genetics and epigenetics of acromegaly [6]. More than 95% of pituitary adenomas occur sporadically, and only a small number is based on already known germline mutations. Although several susceptibility loci have been explored, the genetic etiology of somatotropinomas remains quite diverse and largely unknown. It is challenging to pinpoint genetic mutations that are both frequent and specifically lead to acromegaly. Furthermore, many GH-secreting pituitary adenomas harbor chromosomal alterations such as somatic copy number alterations at extensive sites across the genomes. However, epigenetic mechanisms also appear to play a very important role, and Henriques et al. dedicate an excellent review to current knowledge regarding microRNAs (miRNAs) in acromegaly, addressing their mechanisms of action, their potential role as biomarkers in pathological processes, specific miRNAs associated with acromegaly tumorigenesis as well as resistance to first-generation somatostatin analogs [7]. Several differentially expressed miRNAs are associated with proliferation, invasion, size, and even tumor suppression. Therefore, these promising non-coding RNA snippets may not only elucidate the principles of tumor formation but may also yield therapeutic targets or at least allow better prediction models to be built in the distant future. However, there are still many questions left unanswered and further research is needed before miRNAs are introduced into clinical practice.

Given the etiologically diverse background of somatotroph tumors, it is not surprising that there are several histologically different entities with their own distinct features. Akirov and colleagues diligently collated the published knowledge of histopathological and clinical features of the various entities that lead to GH excess and acromegaly [8]. In another review, Akirov et al. lay down the fundamental aspects of biochemical diagnosis of acromegaly, peering into the role of GH and IGF-I in establishing the diagnosis as well as their function as follow-up biomarkers of disease activity [9]. Especially, the discrepancy between random GH or GH nadir and IGF-I deserves further study since analytical as well as biological and pharmacological aspects factor into this topic [10].

The prognosis of acromegaly depends on several factors [11,12], and it can be assumed that an early diagnosis is associated with a more favorable starting point. A very fascinating paper by Jill Sisco and Aart van der Lely demonstrates both a clinician’s and a patient’s perspective [13]. The former is a patient with acromegaly herself and president of the Acromegaly Community Patient’s Advocacy Organization, the latter a renowned endocrinologist who has participated in consensus conferences and guideline task forces on how to diagnose and manage acromegaly. The personal attachment of both authors to this subject is almost palpable, making it a very interesting and enjoyable read.

The treatment of acromegaly primarily consists of tumor removal, ideally by an experienced neurosurgeon. Tumor size represents a major prognostic factor in terms of surgical cure. As most cases present with macroadenomas, the overall remission rate after surgery is only about 50% [14]. This means that one in two patients will need further treatment, and pharmacological therapy is usually initiated first [15]. In a comprehensive review, Sahakian et al. recapitulate the medical treatment of pituitary tumors and summarize the fundamental aspects of approved and emerging drugs in the treatment of growth-hormone-secreting tumors [16]. The principal substance class used in the medical treatment of acromegaly are somatostatin analogs (SSA; sometimes also referred to as somatostatin receptor ligands: SRL), chemically resembling endogenous somatostatin and effectively inhibiting growth hormone secretion in a significant proportion of patients. Over many years, octreotide and lanreotide were the only agents available, but a few years ago, pasireotide was approved as a third SSA but second line option that can induce disease control in some of the previously uncontrolled patients. This led to a differentiation of SSA in first- and second-generation compounds. By broadening the therapeutic armamentarium, the need for better prediction models has intensified. Several research groups have advocated the use of biomarkers to identify therapeutic responses ideally even before starting treatment, thereby allowing for a more targeted therapeutic approach [17,18]. Gil et al., for instance, analyzed resistance against SSA and the expression of genes related to E-cadherin loss and epithelial–mesenchymal transition [19]. They found a heterogenous expression pattern, but also an indication of a role of *RORC* in SSA response with a higher probability of complete response in pre-treated patients expressing high levels of *RORC*. On the other hand, *SNA1* was associated with tumor invasion and poor SSA response. Based on these and other data, the same group went on to sketch a proof of concept on how data mining analyses can aid in developing targeted treatment approaches in acromegaly. Another group, in turn, presents a study that focused on a *gsp* mutation status in 136 patients with acromegaly and the potential to predict treatment response in first-generation SSA [20], finding that long-term response did not differ between patients with wildtype versus mutated *gsp*. However, mutated-*gsp* growth-hormone-secreting adenomas have a higher expression of somatostatin receptor subtype (SSTR) 5, which is believed to be the driving mechanism of pasireotide efficacy. It would therefore be interesting to see whether patients harboring *gsp* mutations respond more favorably to pasireotide. On the other hand, the role of SSTR5 in acromegaly patients responsive to pasireotide remains controversial [21]. Adding to this debate, Amarù and colleagues present data derived from the incubation of cultured somatotroph tumor cells and rat GH4C1 cells, with a combination of pasireotide, octreotide, and a SSTR2-selective antagonist demonstrating a reversal of the antisecretory effects of both SSAs after SSTR2 antagonism [22]. Furthermore, no synergistic effects were observed after the application of both SSAs. This is surprising, as data from prior animal studies demonstrated the synergistic effects of combining octreotide and pasireotide, potentially increasing efficacy, while improving tolerability [23]. Recent data from a phase I study also appear to support this finding [24]. There is a need for further research into this highly relevant clinical topic. Hopefully, we look forward to further exciting research that sheds more light on this deeply fascinating disease.
